# Differentiation of malignant cervical lymphadenopathy by dual-energy CT: a preliminary analysis

**DOI:** 10.1038/srep31020

**Published:** 2016-08-08

**Authors:** Liang Yang, Dehong Luo, Lin Li, Yanfeng Zhao, Meng Lin, Wei Guo, Chunwu Zhou

**Affiliations:** 1 Radiology Department, Cancer Hospital, Chinese Academy of Medical Sciences & Peking Union Medical College, Beijing, 100021, China

## Abstract

The accurate diagnosis of malignant cervical lymphadenopathy remains challenging. In this study, we determined the value of quantitative parameters derived from dual-energy computed tomography (DECT) for differentiating malignant cervical lymphadenopathy caused by thyroid carcinoma (TC), salivary gland carcinoma (SC), squamous cell carcinoma (SCC) and lymphoma. We retrospectively analysed 92 patients with pathologically confirmed cervical lymphadenopathy due to TC, SC, SCC and lymphoma. All patients received a DECT scan before therapy. Using GSI (gemstone spectral imaging) Volume Viewer software, we analysed the enhanced monochromatic data, and the quantitative parameters we acquired included the iodine concentration (IC), water concentration (WC) and the slope of the spectral HU curve (λ_HU_). One-way ANOVA showed significant differences in the IC and λ_HU_ among different groups (*P* < 0.05). Post-hoc pairwise comparisons demonstrated the IC and λ_HU_ of TC group were significantly higher than those of SC, SCC and lymphoma groups (*P* < 0.05). In addition, the IC and λ_HU_ of SC group were significantly higher than those of the SCC and lymphoma groups (*P* < 0.05). Other comparisons of IC and λ_HU_ values showed no significant differences (*P* > 0.05). The quantitative parameters derived from DECT were useful supplements to conventional computed tomography images and were helpful for distinguishing different malignant cervical lymphadenopathies.

The neck is rich in lymph nodes, with approximately 40% of the total lymph nodes in the human bod^1^. Cervical lymph node lesions are variable, and the malignancy rate is over 50%^1^,^2^. Lymph node metastatic carcinoma and lymphoma are the most common cervical malignant lesions, with cervical lymph node metastatic carcinoma accounting for approximately 3/4 of all malignant neck tumours. Most cervical malignant lesions (85%) include primary lesions located in the head and neck, and the most common lesions are nodal metastases of thyroid carcinoma (TC), salivary gland carcinoma (SC) and squamous cell carcinoma (SCC)^3^,^4^. Therefore, it would be valuable to identify the nature and types of cervical lymph node lesions before selecting clinical treatments^5^,^6^. The typical malignant cervical lesions of lymph nodes are not difficult to diagnose with imaging, but some lesions are not typical, and imaging features can overlap, which makes it difficult for health professionals to establish a diagnosis using conventional imaging^7^. Imaging of cervical lymphadenopathy is a challenge owing to the difficulty of diagnosing malignant lymph nodes accurately before treatment. New imaging techniques are needed to integrate both the morphological and functional changes of lymph node lesions for better evaluation and treatment.

Dual-energy computed tomography (DECT) with the latest gemstone detector allows for reconstruction of virtual monochromatic images and material decomposition images^8^,^9^,^10^,^11^,^12^ and provides multiple quantitative parameters that have been used for the diagnosis of lymph node metastasis for several types of tumours^12^,^13^,^14^,^15^. However, to our knowledge, the value of DECT for the preoperative diagnosis of cervical nodal metastasis in patients with different malignant carcinomas has not been well evaluated. The purpose of this study was to investigate the application of DECT to identify different malignancies in cervical lymph nodes.

## Results

### Patients’ characteristics

The pathological diagnoses of all malignant cervical lymphadenopathies are listed in Table 1.

### Differences in sex and age between different lymph node groups

The incidence rates of SC, SCC, and lymphoma in males were higher than those in females, but the opposite trend occurred in the TC group, and the differences were significant (*P* < 0.05). The differences in age between the different lymph node groups were significant (*P* < 0.05) (Table 2).

### Comparison of DECT quantitative parameters between different lymph node groups

**Figure 1:**
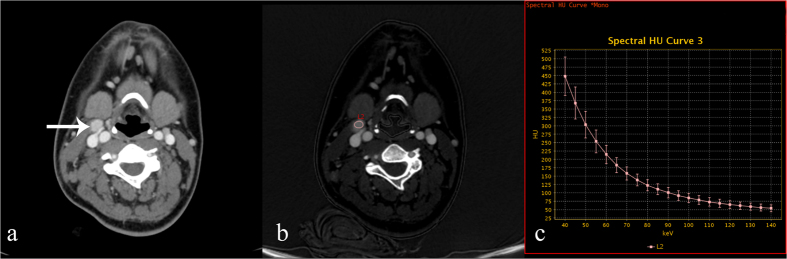
70 keV contrast-enhanced monochromatic and GSI images in a 22-year-old woman with papillary throid carcinoma. GSI images show that IC and λ_HU_ in metastatic lymph nodes are significantly higher than those of SC, SCC and lymphoma groups. (**a**) An axial CT image shows a right level II metastatic enlarged lymph node (white arrow). (**b**) An iodine-based material-decomposition image shows that the IC of the metastatic lymph nodes was 45.05 × 10^2^ μg/cm^3^ (L2: area, 42.52 mm^2^). (**c**) The graph shows the spectral HU curve of a metastatic lymph node; λ_HU_ is 6.95.

**Figure 2:**
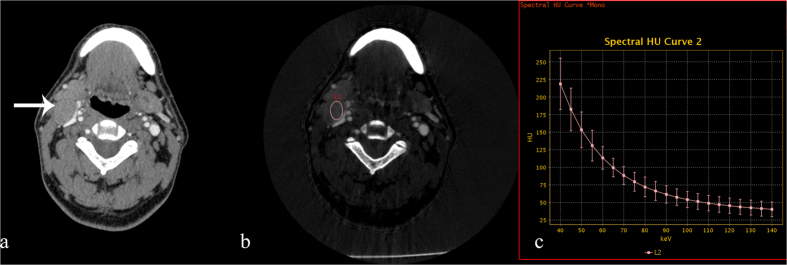
70 keV contrast-enhanced monochromatic and GSI images from a 53-year-old man with mucoepidermoid carcinoma of the minor salivary glands. GSI images show that the IC and λ_HU_ values in metastatic lymph nodes are significantly lower than that in the TC group and higher than those in the SCC and lymphoma groups. (**a**) An axial CT image shows a right level II metastatic enlarged lymph node (white arrow). (**b**) An iodine-based material-decomposition image shows that the IC in metastatic lymph nodes is 23.04 × 10^2^ μg/cm^3^ (L2: area, 133.49 mm^2^). (**c**) The graph shows the spectral HU curve of a metastatic lymph node; λ_HU_ is 3.14.

**Figure 3:**
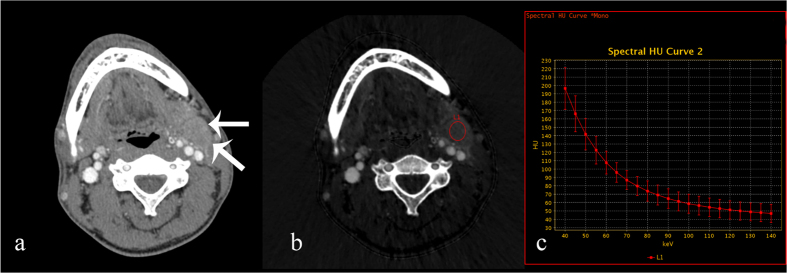
70 keV contrast-enhanced monochromatic and GSI images of a 28-year-old man with nasopharyngeal squamous cell carcinoma. GSI images show that the IC and λ_HU_ of metastatic lymph nodes are significantly lower than those of the TC and SC groups. (**a**) An axial CT image shows a left level II metastatic enlarged lymph node (white arrow). (**b**) An iodine-based material-decomposition image shows that the IC in metastatic lymph nodes is 18.97 × 10^2^ μg/cm^3^ (L1: area, 398.57 mm^2^). (**c**) The graph shows the spectral HU curve of a metastatic lymph node; λ_HU_ is 2.57.

**Figure 4:**
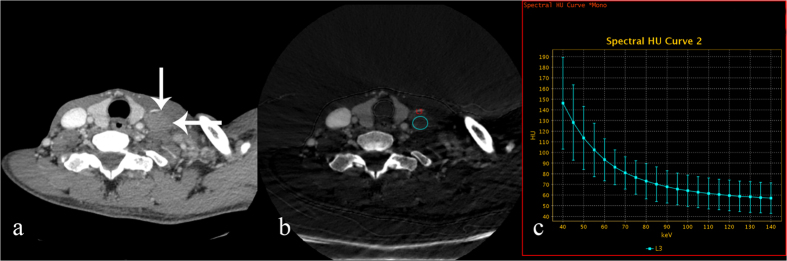
70 keV contrast-enhanced monochromatic and GSI images of a 52-year-old man with diffuse large B-cell lymphoma. GSI images show that the IC and λ_HU_ in metastatic lymph nodes are significantly lower than those in the TC and SC groups. (**a**) An axial CT image shows a left level IV metastatic enlarged lymph node (white arrow). (**b**) An iodine-based material-decomposition image shows that the IC in the metastatic lymph nodes is 11.57 × 10^2^ μg/cm^3^ (L3: area, 137.57 mm^2^). (**c**) The graph shows the spectral HU curve of the metastatic lymph node; λ_HU_ is 1.57.

**Figure 5:**
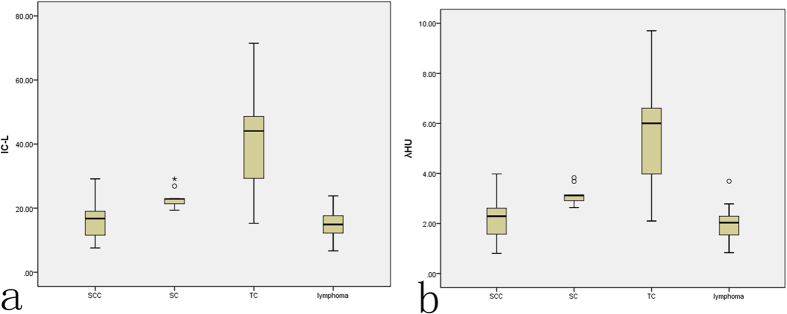
Box and whisker plots of the IC (**a**) and λ_HU_ (**b**) values for metastatic SCC, SC and TC lymph nodes and lymphoma.

The IC values for TC, SC, SCC, and lymphoma were 38.18 ± 15.92 × 10^2^ μg/cm^3^, 23.01 ± 3.15 × 10^2^ μg/cm^3^, 16.00 ± 5.31 × 10^2^ μg/cm^3^, and 14.66 ± 4.17 × 10^2^ μg/cm^3^, respectively. The λ_HU_ values for TC, SC, SCC, and lymphoma were 5.22 ± 2.15, 3.12 ± 0.41, 2.16 ± 0.75, and 1.95 ± 0.64, respectively. The differences in both IC and λ_HU_ values among the different groups were significant (*F* = 37.41 and 38.12, respectively, *P* < 0.05). Post hoc pairwise comparisons of the IC and λ_HU_ demonstrated significant differences between the TC group and the SC, SCC or lymphoma groups (*P* < 0.05) and between the SC group and the SCC or lymphoma groups (*P* < 0.05), whereas no significant difference was observed between the SCC and lymphoma groups (*P* > 0.05) (Tables 3 and 4, Figs 1, 2, 3, 4, 5). The medians of the WC in the TC, SC, SCC, and lymphoma groups were 1036.22 (1026.298–1042.14) mg/cm^3^, 1032.25 (1025.03.31–1036.17) mg/cm^3^, 1034.63 (1027.68–1038.61) mg/cm^3^, and 1034.08 (1031.21–1041.26) mg/cm^3^, respectively, and no significant difference in the WC was observed between the different groups (*H* = 0.39, *P* > 0.05) (Table 5).

## Discussion

The qualitative diagnosis of malignant lesions in the cervical lymph node directly affects the selection of treatment options and the patient prognosis^16^,^17^. Previous imaging diagnoses of lymph node lesions have mainly depended on changes in lesion morphology, such as the size, shape, and necrotic area^18^. Although malignant lesions in different cervical lymph nodes manifest certain imaging characteristics, and typical cases are not difficult to diagnose, some atypical cases cannot be diagnosed accurately because conventional imaging techniques cannot provide specificity and sensitivity simultaneously. The accurate diagnosis of cervical lymph nodes malignancies has always been a difficult problem for radiologists^19^. Combining the changes in both morphology and function could allow for better evaluation of the lesions in lymph nodes and lead to the development of new diagnostic imaging techniques.

Conventional computed tomography (CT) imaging uses X-ray beams generated from a tube containing a broad energy spectrum^20^,^21^,^22^. The polychromaticity of X-rays causes beam hardening artefacts and an averaging attenuation effect in images^23^,^24^. In contrast, DECT uses a single tube with fast kilovolt dynamic switching between 80- and 140-kVp X-rays on adjacent views during a single rotation, which creates two sets of coherent energy information. The two sets of energy information are aligned in projection space, and sets of monochromatic images in a range of 40–140 keV are generated through projection-based reconstruction^25^,^26^,^27^,^28^,^29^. DECT provides quantitative and qualitative analysis of the tissue^30^,^31^, and has been widely used in the diagnosis of diseases in various systems in the human body^32^,^33^,^34^,^35^.

Using the two-material decomposition technique, the unique linear attenuation coefficients obtained by monochromatic imaging at two different energies can be used to discriminate between different materials (calcium, iodine, water, fat, etc.) in DECT, which can provide relatively accurate quantitative data about those materials^36^. The different material decomposition images can be reconstructed from monochromatic data, such as iodine (water), water (iodine), and iodine (calcification). Because iodine is the major component of the CT contrast medium, measuring the iodine concentration with iodine (water) imaging reflects the enhancement of lesions^37^.

This study showed that the IC values of different groups were significantly different, and pairwise comparisons demonstrated that the IC value of metastatic lymph nodes in the TC group was significantly higher than those of other metastatic carcinoma and lymphoma groups (*P* < 0.05), which may be due to the rich blood supply of TC metastatic lymph nodes and the iodine-absorbing features of thyroid tissue. Regarding the biological behaviour, SC can be classified into low-grade and high-grade malignant types. Lymph node metastasis rarely occurs for the low-grade malignant type, whereas 50–80% of high-grade malignant types show lymph node metastasis. In this study, the IC value of metastatic lymph nodes in the SC group was lower than that of the TC group, which may be related to the low differentiation, higher degree of malignancy, and richer blood supply of SC with lymph node metastasis. This study showed that the IC values of the lymphoma and SCC groups were not significantly different. This result might be caused by the replacement and destruction of all or part of the normal lymph node structures by the invading tumour cells and the inconspicuous angiogenesis that occurs in lymphoma. However, the invasion of SCC starts from the marginal sinus of the lymph node cortex; then, it infiltrates the medulla and leads to lymphatic obstruction, followed by necrosis of the medulla region, and it manifests as a small central low density region composed of necrotic tissue, keratin, fibrous tissue, interstitial oedema and surviving tumour cells, which might be responsible for the lower IC values.

The λ_HU_ reflects attenuation in HU values of tissue or material across the 40~140 keV range. The linear attenuation coefficient of substances declines with increased keV, but each material has a different rate of decline, i.e., the λ_HU_ can present energy decay and is determined by the physical and chemical nature of the material itself. This principle is exploited to enhance the contrast between different tissues at any selected keV^38^,^39^,^40^,^41^. The λ_HU_ value of the TC group was 5.45 ± 1.95, which was significantly higher than that of SC, SCC or lymphoma group (*P* < 0.05). The λ_HU_ value of nodes from the SC group was 3.12 ± 0.41, which was the second highest value and significantly higher than those of the SCC and lymphoma groups (*P* < 0.05). The differences in λ_HU_ values between the SCC and lymphoma groups were not significant (*P* > 0.05), which was possibly related to the variety of pathological types in the lymphoma group; therefore, no stratification analysis of the lymphoma group was conducted. The WC demonstrated no significant difference between the different groups, which showed that the difference in the WC was not as sensitive as differences in the IC and λ_HU_.

Studies have shown that many techniques, including MR diffusion, spectroscopy, MRI perfusion, and contrast enhanced ultrasound, have certain effects on diagnosing different lymphadenopathies, but these approaches are restricted in clinical practice by high costs, long scanning times, poor display of small lesions, artefacts, frequent false-positive findings, and other detrimental factors^42^,^43^,^44^,^45^,^46^,^47^. CT can complete one cervical scan in one breath-hold with fast scan and get high density resolution imaging, and it is the most frequently used method for detecting cervical lymphadenopathies. In our study, the IC and λ_HU_ values for the TC, SC, SCC, and lymphoma groups gradually declined, and the differences, except that between the SCC and lymphoma group, were significant. Therefore, these values could be used as auxiliary indexes of diagnosis. This study also showed that SC, lymphoma and SCC were more common in men, and the incidence of SCC was much higher than the incidence of other tumours in men. Moreover, the ages among different groups were significantly different (*P* < 0.05). Previous studies have shown that age and sex are important factors for classifying neck masses^1^,^17^. In general, neck masses in children and young adults are more inflammatory than congenital and neoplastic diseases and are only found occasionally^4^. However, masses in older adults are often neoplastic^48^. In our study, the individuals in the SCC group were older than in the groups with other malignant lymphadenopathies, particularly the lymphoma group. Combining the IC and λ_HU_ with age and sex could help differentiate lymphadenopathies in clinical practice.

Some limitations were present in this study. First, owing to the number and type of cases, hierarchical analysis was not conducted among different groups. Second, the sample size of the SC group was small. Furthermore, patients with lymphoma and partial metastatic lymph nodes were not treated surgically. Even for some patients with SCC, the biopsy node that the histological findings were based on did not exactly match the node that we selected and measured using the CT post-processing workstation. The imaging diagnosis of a malignant node was based on recognized criteria, and only the largest node rather than all abnormal nodes was evaluated. The number of nodes could have increased with evaluation of more than one node in each patient, but this change would have produced dependency issues regarding the influence of multiple nodes per patient. Therefore, this approach was not used.

Our results confirmed that quantitative parameters derived from DECT were a useful supplement to conventional CT images and were helpful for distinguishing different malignant cervical lymphadenopathies. Using these quantitative parameters in the preoperative evaluation of cervical malignant lymphadenopathies might help radiologists avoid subjective bias related to experience and increase diagnostic confidence. These experiments therefore offered important information for diagnosing different cervical malignant lymphadenopathies. Next, the diagnostic efficiency of classifying different types of lymph nodes should be studied, which will provide a basis for better diagnosis of cervical lymph node lesions.

## Materials and Methods

### Inclusion and exclusion criteria

This prospective study was approved by the institutional review board at the Cancer Hospital of the Chinese Academy of Medical Sciences. Informed consent was obtained from all participants. This study was conducted in accordance with the Declaration of Helsinki.

The study was performed from January 2014 to June 2015 on consecutive patients with enlarged neck lymph nodes that clinically suggested malignancy. The inclusion criteria were as follows: (1) pathological findings included metastatic lymph nodes due to TC, SC, SCC and lymphoma; (2) lesions >0.5 cm at the shortest diameter; and (3) the patient was not receiving any chemotherapeutic or radiotherapeutic treatments. Overall, 92 patients (mean age, 46.85 ± 15.43 years; age range, 12–73 years; 27 women and 65 men) were ultimately enrolled in the study. Upon examination, 25 patients were excluded from this study. The exclusion criteria were as follows: (1) benign lesions confirmed by biopsy or postoperative pathological diagnosis for 20 cases (7 cases of lymphadenitis, 5 cases of reactive lymphaden hyperplasia, 2 cases of giant lymph node hyperplasia, and 6 cases of lymph node tuberculosis) and (2) metastatic lymph nodes due to non-primary neck malignant tumours (lung cancer; gastric cancer; unknown primary tumour; and prostate, cervical, and ovarian cancers, including 6, 3, 3, 1, 1, and 1 cases, respectively).

### CT examination

All patient pathologies were confirmed by histological biopsy or pathological examinations. Then, patients received DECT (Discovery CT750 HD, GE Healthcare, Waukesha, Wis) in gemstone spectral imaging mode before treatment. The scanning parameters were as follows: GSI-17 protocol (manufacturer number), helical mode, axial plane with coverage from mid orbits to the clavicular heads, collimator of 20 mm, slice thickness of 1.25 mm, slice interval of 0.8 mm, pitch of 0.984, tube current of 550 mA, tube voltage fast switching between 80 kVp and 140 kVp with a cycle of 0.5 ms and an FOV (field of view) of medium body. All patients were intravenously injected with contrast media (Ultravist 300; Bayer Pharma AG, Leverkusen, Germany) using a power injector with a rate of 2.5 ml/s, and volume of 1.5 ml/kg (85–100 ml). The scan acquisition was started after a delay of 30 s.

### Image analysis

The original data acquired were reconstructed into monochromatic images. The reconstructed images were sent to a post-processing workstation (Advantage Workstation 4.6, GE Healthcare, Milwaukee, WI). GSI (gemstone spectral imaging) Volume Viewer software on the workstation was used to analyse the enhanced monochromatic data and acquire the quantitative parameters. For the axial image, a radiologist with 10 years of experience in CT diagnosis of head and neck tumours selected the maximum level of the metastatic lymph node and selected the region of interest (ROI). The ROI was placed at the middle level of the lesions, and the range was not less than 1/2 of the same level of substantial mass and necrotic tissue. Voids, calcification and blood vessels were avoided. When more than one enlargement was present, cervical lymph nodes were located, and the largest metastatic lymph node was chosen. The quantitative parameters were measured or calculated, including the iodine concentration of the lesion (IC), water concentration of the lesion (WC), and slope of the spectral HU curve (λ_HU_), which was calculated as the difference between the CT value at 40 keV and that at 90 keV divided by the energy difference (50 keV): λ_HU_ = (CT_40keV_ − CT_90keV_)/50.

### Statistical analysis

A database was created using Microsoft Excel, and statistical analyses were performed with the SPSS 19.0 (SPSS Inc., Chicago, IL) statistical software package. Quantitative data with a Gaussian distribution are presented as means ± standard deviations (*X* ± *S*), and quantitative data with non-normal distributions are presented as medians (inter-quantile ranges) or *M* values (*P*_25_–*P*_75_). The normality and homogeneity of variance among all measurement data were analysed. If the data exhibited a normal distribution and homogeneity of variance, one-way ANOVA was used, and an inter-group comparison was performed with the LSD method. If the data did not have a normal distribution or homogeneity of variance, the Kruskal-Wallis H test was used, and inter-group comparisons were performed with the Tamhane method. Gender proportions were compared using a chi-square (*χ*^*2*^) test, and α = 0.05 was defined as the level of significance. A value of *P* > α indicated no statistically significant difference.

## Additional Information

**How to cite this article**: Yang, L. *et al.* Differentiation of malignant cervical lymphadenopathy by dual-energy CT: a preliminary analysis. *Sci. Rep.* **6**, 31020; doi: 10.1038/srep31020 (2016).

## Figures and Tables

**Table 1:** Pathological diagnosis of all patients.

Diagnosis	Case number (n = 92)
Thyroid carcinoma	18 (19.57%)
Papillary carcinoma	18 (19.57%)
Salivary carcinoma	9 (9.78%)
Adenoid cystic carcinoma	2 (2.17%)
Mucoepidermoid carcinoma	1 (1.09%)
Epithelial-myoepithelial carcinoma	1 (1.09%)
Nonspecific adenocarcinoma	1 (1.09%)
Metastasizing pleomorphic adenoma	1 (1.09%)
Ductal adenocarcinoma	2 (2.17)%
Basal cell adenocarcinoma	1 (1.09%)
SCC	36 (39.13%)
Nasopharyngeal carcinoma	13 (14.13%)
Tonsillar carcinoma	1 (1.09%)
Pharyngeal carcinoma	8 (8.70%)
Hypopharyngeal carcinoma	4 (4.35%)
Tongue carcinoma	4 (4.35%)
Esophageal carcinoma	2 (2.17%)
Oropharyngeal carcinoma	4 (4.35%)
Lymphoma	29 (31.52%)
Mantle cell lymphoma	1 (1.09%)
Follicular Lymphoma	4 (4.35%)
Diffuse large B-cell lymphoma	14 (15.22%)
Peripheral T-cell lymphoma	1 (1.09%)
Nodular sclerosis Hodgkin lymphoma	3 (3.26%)
Mixed cellularity Hodgkin lymphoma	2 (2.17%)
Lymphocyte-rich Hodgkin lymphoma	1 (1.09%)
Lymphocyte depletion Hodgkin lymphoma	1 (1.09%)
Extranodal nasal-type natural killer/T-cell lymphoma	1 (1.09%)
Peripheral T-cell lymphoma	1 (1.09%)

TC is the abbreviation of thyroid carcinoma. SC is the abbreviation of salivary carcinoma. SCC is the abbreviation of squamous cell carcinoma.

**Table 2:** Different malignant cervical lymphadenopathies according to patient age and sex.

	Thyroid carcinoma	Salivary carcinoma	SCC	Lymphoma	*Statistics Value*	*P*
Sex (M/F)	7/11	6/3	34/2	18/11	21.91^a^	0.00*
Age (y)	45.68 ± 16.67	52.44 ± 12.05	52.19 ± 12.54	39.90 ± 16.49	4.37^b^	0.01*

^a^Indicates the *χ*^*2*^value.

^b^Indicates the *F* value.

^*^Indicates statistical significance.

**Table 3:** Differences in IC and λ_HU_ between different lymph node groups.

	Thyroid carcinoma	Salivary carcinoma	SCC	Lymphoma	*F*	*P*
IC (×10^2^ μg/cm^3^)	39.85 ± 14.57	23.01 ± 3.15	16.00 ± 5.31	14.66 ± 4.17	48.31	0.00*
λ_HU_	5.45 ± 1.95	3.12 ± 0.41	2.16 ± 0.75	1.95 ± 0.64	49.16	0.00*

^*^Indicates statistical significance.

**Table 4:** Post hoc pairwise comparisons of IC, λ_HU_ (*LSD-t* test).

Comparative group (R_A_ vs R_B_)	IC (100 μg/cm^3^)		λ_HU_	
	Difference of means 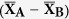	*P*	Difference of means 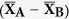	*P*
TC vs SC	16.84	0.00*	2.33	0.00*
TC vs SCC	23.86	0.00*	3.30	0.00*
TC vs Lymphoma	25.19	0.00*	3.45	0.00*
SC vs SCC	7.01	0.02*	0.96	0.02*
SC vs Lymphoma	8.35	0.01*	1.17	0.01*
SCC vs Lymphoma	1.34	0.49	0.21	0.43

^*^Indicates statistical significance.

**Table 5:** Differences in WC between different lymph node groups.

	TC	SC	SCC	Lymphoma	*H*	*P*
WC (mg /cm^3^)	1036.22 (1026.298~1042.14)	1032.25 (1025.03~1036.17)	1034.63 (1027.68~1038.61)	1034.08 (1031.21~1041.26)	0.39	0.95
